# Compliance With the WHO Safe Childbirth Checklist at a Tertiary Care Hospital in Pakistan: A Closed-Loop Clinical Audit

**DOI:** 10.7759/cureus.87853

**Published:** 2025-07-13

**Authors:** Mostafa Ahmed Abdellah Ahmed, Bilal Fattani, Bilal Masood, M Khaliq

**Affiliations:** 1 General Surgery, Frimley Health NHS Foundation Trust, Frimley, GBR; 2 Surgery and Allied, Jinnah Medical and Dental College, Karachi, PAK; 3 Community Medicine, Bolan Medical College, Quetta, PAK; 4 Pathology, Federal Postgraduate Medical Institute, Lahore, PAK; 5 Pathology, University of Health Sciences, Lahore, PAK

**Keywords:** clinical audit, maternal safety, neonatal care, pakistan, quality improvement, safe childbirth checklist

## Abstract

Background: The World Health Organization (WHO) Safe Childbirth Checklist (SCC) is a 29-item instrument aimed at standardizing the key maternal and neonatal safety practices. However, there is a scarcity of data on real-world SCC compliance in Pakistan. The purpose of the study was to determine the baseline adherence to the WHO SCC at the Federal Postgraduate Medical Institute in Lahore, Pakistan, carry out an educational intervention, and gauge improvements after the intervention.

Methods: A prospective, closed-loop clinical audit was performed between December 1, 2023, and February 15, 2024. A pre-intervention cycle (Cycle 1) was used to determine baseline SCC adherence by observing 60 consecutive deliveries. An educational workshop, involving item-by-item demonstrations, team-communication role-plays, and the placement of laminated checklists at every pause point, was conducted among obstetricians, midwives, and nurses on December 31, 2024. Post-intervention compliance was then audited by a second cycle (Cycle 2) that sampled 60 consecutive deliveries. All 29 SCC items were noted in terms of “Yes/No” completion by trained auditors at the four pause points. Quantitative tests were carried out to determine the change in complete and phase-by-phase compliance between the study periods.

Results: The compliance from completed checklists increased from Cycle 1 by 35 (58.3%) to Cycle 2 by 47 (78.3%). The largest improvements were seen on admission from 40 (70.0%) to 50 (86.7%), before pushing or cesarean from 35 (58.3%) to 52 (86.7%), within one hour after birth from 32 (53.3%) to 51 (85.0%), and before discharge from 33 (55.0%) to 50 (80%). The largest item-specific increases were seen in breastfeeding with skin-to-skin contact, from 38 (63.3%) to 49 (81.7%), and the discharge-planning item, namely family-planning counseling, from 33 (55.0%) to 43 (71.7%).

Conclusion: The well-organized educational program had a significant effect on WHO SCC compliance in the tertiary care hospital in Pakistan. To achieve lasting change and minimize avoidable maternal and neonatal harm, the audit-feedback cycles and leadership involvement will be needed on a regular basis, as well as the incorporation of the SCC into regular clinical practice.

## Introduction

Maternal and infant morbidity and mortality remain urgent public health concerns in Pakistan, where complications during birth contribute to many preventable deaths [1]. In 2021, the proportion of maternal mortality was about 140 per 100,000 live births, while the neonatal mortality rate constituted 42 per 1,000 live births - both significantly higher than global targets [2]. Intrapartum and immediate postnatal care thus should be of the highest quality, especially in tertiary care settings where high-risk pregnancies are handled. The World Health Organization (WHO) developed the Safe Childbirth Checklist (SCC) in 2015 to standardize best safety practices and minimize errors [3]. The SCC includes 29 evidence-based items that are grouped into four critical pauses: on admission, before pushing or cesarean section (C-section), within one hour after birth, and before discharge [4].

The items remind teams to verify interventions, including partograph initiation, early antibiotic prophylaxis, magnesium sulfate for treating pre-eclampsia, early breastfeeding, and family-planning advice [5]. Low-pilot implementations in resource-limited settings have demonstrated that the use of the SCC can improve adherence to lifesaving practices, increasing compliance from about 10-15 items at baseline to more than 20 items after specific training [6]. Even though the potential of such an approach has been proven, there is little systematic auditing of SCC compliance in Pakistani hospitals. Issues such as irregular staff training, power dynamics, and checklist fatigue could undermine the implementation and sustainability of checklists. Additionally, maintaining the initial gains requires routine audit-feedback loops, executive sponsorship, and incorporation into standard operating procedures (SOPs) [7].

The current study describes a closed-loop clinical audit of WHO SCC implementation at the Federal Postgraduate Medical Institute, a tertiary care hospital in Lahore that performs more than 1,200 deliveries every year. We aimed to measure baseline adherence to each of the 29 items during 60 consecutive deliveries (pre-intervention), provide a structured educational intervention to obstetric teams, and then re-measure compliance during 60 further deliveries (post-intervention) to outline unaddressed gaps and shape the further implementation of the SCC into routine practice, thereby enhancing maternal and neonatal safety in Pakistan.

## Materials and methods

This was a prospective, closed-loop clinical audit performed at the Federal Postgraduate Medical Institute between December 1, 2023, and February 15, 2024, under approval number 331-23. All women who were in the labor room due to spontaneous labor or elective C-section during this time were included; those who had elective cesareans outside the labor room were excluded. The audit had two cycles. Cycle 1 focused on the observation of 60 consecutive deliveries between December 1 and December 30, 2023, to establish a baseline of compliance. The sample size of 60 deliveries per cycle was chosen according to the standards of audit methodology to ensure a significant comparison within the study period. Following Cycle 1, an educational intervention was held on December 31, 2023, which comprised a two-hour practical lesson covering the rationale of the WHO SCC, step-by-step modeling, team communication role-plays, and visual supports in the form of laminated SCC posters at every pause point. Cycle 2 observed 60 consecutive deliveries between January 1 and February 15, 2024, to evaluate compliance following the intervention.

Certified auditors utilized a structured observation form based on the WHO SCC, and the completion of the 29 items was noted as either "Yes/No" at the four predetermined pause moments [8]. The entire checklist was availed in both cycles. Data were analyzed using IBM SPSS Statistics v26.0 (IBM Corp., Armonk, USA). We determined the number of compliant cases (n) out of 60 and calculated the percentages for each item. Statistical comparisons were made to validate the respective phase-wise and total compliance across cycles.

## Results

The total deliveries audited were 120 (60 in the pre-intervention Cycle 1 and 60 in the post-intervention Cycle 2). Complete checklist compliance with all 29 SCC items was 35/60 (58.3%) and improved to 47/60 (78.3%) after the educational intervention. Table 1 demonstrates item-wise compliance with sample sizes and percentages for both cycles.

**Table 1:** Compliance rates for all 29 SCC items SCC, Safe Childbirth Checklist; ARVs, Antiretrovirals; C-section, Cesarean Section; n, Number of compliant cases

Item No.	Checklist Item	Cycle 1 (n/60, %)	Cycle 2 (n/60, %)
On Admission			
1	Referral need?	55/60 (91.7%)	59/60 (98.3%)
2	Partograph started?	52/60 (86.7%)	58/60 (96.7%)
3	Antibiotics if indicated?	48/60 (80.0%)	56/60 (93.3%)
4	Magnesium sulfate if indicated?	47/60 (78.3%)	54/60 (90.0%)
5	Nevirapine if indicated?	45/60 (75.0%)	53/60 (88.3%)
6	Encourage birth companion?	39/60 (65.0%)	50/60 (83.3%)
7	Hand‐cleaning supplies ready?	42/60 (70.0%)	55/60 (91.7%)
8	Mother/companion instructed to call for help if danger signs?	35/60 (58.3%)	46/60 (76.7%)
Before Pushing/C-Section			
9	Antibiotics if indicated?	44/60 (73.3%)	53/60 (88.3%)
10	Magnesium sulfate if indicated?	43/60 (71.7%)	52/60 (86.7%)
11	Essential supplies for mother prepared?	40/60 (66.7%)	50/60 (83.3%)
12	Essential supplies for baby prepared?	38/60 (63.3%)	49/60 (81.7%)
13	Assistant identified and ready?	30/60 (50.0%)	40/60 (66.7%)
Within One Hour After Birth			
14	Check for abnormal maternal bleeding?	50/60 (83.3%)	57/60 (95.0%)
15	Newborn antibiotics if indicated?	47/60 (78.3%)	55/60 (91.7%)
16	Magnesium sulfate for mother if indicated?	46/60 (76.7%)	54/60 (90.0%)
17	Baby referral arranged if needed?	45/60 (75.0%)	56/60 (93.3%)
18	Baby antibiotics if indicated?	44/60 (73.3%)	54/60 (90.0%)
19	Special baby care if indicated?	43/60 (71.7%)	53/60 (88.3%)
20	Baby ARVs if indicated?	41/60 (68.3%)	52/60 (86.7%)
21	Breastfeeding and skin‐to‐skin contact initiated?	38/60 (63.3%)	49/60 (81.7%)
22	Mother/companion instructed to call if danger signs?	32/60 (53.3%)	44/60 (73.3%)
Before Discharge			
23	Confirm maternal bleeding controlled?	49/60 (81.7%)	58/60 (96.7%)
24	Continue maternal antibiotics if indicated?	48/60 (80.0%)	59/60 (98.3%)
25	Continue baby antibiotics if indicated?	47/60 (78.3%)	59/60 (98.3%)
26	Confirm baby is feeding well?	46/60 (76.7%)	58/60 (96.7%)
27	If mother HIV-positive, ARVs for mother and baby?	10/60 (16.7%)	22/60 (36.7%)
28	Family-planning counseling offered?	33/60 (55.0%)	43/60 (71.7%)
29	Arrange follow-up and danger-signs advice at discharge?	31/60 (51.7%)	42/60 (70.0%)

The intervention increased the percentage of the item "breastfeeding and skin-to-skin contact initiated" (from 38/60, 63.3% in Cycle 1 to 49/60, 81.7% in Cycle 2) and encouraged the mother or companion to seek help in case of danger signs (from 32/60, 53.3% to 44/60, 73.3%). During the discharge-planning stage, adherence of HIV-positive mothers to six-week antiretrovirals for both themselves and their babies improved from 10/60 (16.7%) to 22/60 (36.7%). Discussing and offering family-planning options to the mother increased from 33/60 (55.0%) to 43/60 (71.7%), and arranging follow-up and advising on danger signs after discharge also increased from 31/60 (51.7%). These item-specific increases point to a significant rise in the care of newborns during early and discharge planning, while the currently still low rate of maternal HIV management uptake indicates the necessity for specific sensitization.

## Discussion

Retrospectively, this closed-loop audit highlights the fact that a targeted educational bundle can significantly improve compliance with the WHO SCC in a low-resource tertiary care facility [8]. The feasibility of this intervention and its impact have been demonstrated in resource-constrained settings, including those described by Semrau et al. in India and Tanzania, where adherence rates increased by 10-25% after the systematic implementation of the WHO SCC [9]. Baseline overall compliance was 35/60 (58.3%), which indicates moderate adoption, as is the case in other low- and middle-income settings [10,11]. The structured team training and easily available checklist prompts were observed, as post-intervention compliance increased by 19.8 percentage points to 47/60 (78.3%).

Among the main positive changes were antibiotic prophylaxis prior to delivery (Items 3 and 9: 80.0%-93.3%, 73.3%-88.3%) and magnesium sulfate administration in the case of pre-eclampsia (Items 4, 10, and 16: 78.3%-90.0%, 71.7%-86.7%, 76.7%-90.0%), which are the most crucial interventions in a setting with a high burden of sepsis [12]. Promotion of a birth companion (65.0%-83.3%) and breastfeeding with skin-to-skin contact (63.3%-81.7%) also experienced significant increases, consistent with findings that these procedures enhance maternal satisfaction and thermoregulation of neonates [13]. The existence of a birth companion has been correlated with shorter labor, fewer C-sections, and higher appearance, pulse, grimace, activity, and respiration (APGAR) scores, as detailed in the WHO recommendations [14]. Nonetheless, low HIV risk perception and local stigma are evidenced by the consistently low uptake of maternal HIV control (Item 27: 16.7%-36.7%), which underscores the necessity of an opt-out screening policy and confidentiality training. Discharge planning (Item 29: 51.7%-70.0%) showed some improvement but remained behind, possibly indicating that the intense postpartum workload and shift changes disrupt the final checklist items. Designating a discharge champion or incorporating electronic reminders might help cover this gap [15]. The addition of digital reminders has been useful in enhancing adherence to discharge protocols, as observed in a study where mobile health (mHealth) checklists were incorporated into postpartum care pathways in Kenya [16].

Analysis by phase showed the most potential for improvement in the "before pushing/C-section" pause point, where compliance increased from a mean of 58.3% to 80.0%. Since this stage is characterized by acute pressures, it is advisable to integrate quick-reference SCC cards into the delivery suites to further entrench real-time utilization [17]. To be sustainable, the audit-feedback cycles need to be frequent, leadership must be involved, and the SCC must become part of the SOPs [18]. Research identifies practical methods for sustaining maternal health interventions, including the incorporation of tools such as the SCC into national SOPs, continuous training, and multidisciplinary ownership [19]. Accountability can be established through monthly multidisciplinary reviews of adherence data, and SCC training can be integrated into new staff orientation to maintain continuity in the face of turnover [20].

However, this study has several limitations. Its single-center nature may limit overall generalizability to other healthcare facilities. The Hawthorne effect may have artificially affected compliance rates due to staff awareness of being observed. The absence of clinical outcomes data prohibits evaluating the impact of the checklist on maternal/newborn health in the real world. Policymakers also face limitations in their understanding of the study due to the absence of cost-effectiveness analysis. Moreover, unmeasured confounding variables (e.g., staffing density or multimodal interventions) may have interfered with the research findings.

## Conclusions

A targeted educational intervention profoundly enhanced the WHO SCC compliance at the Federal Postgraduate Medical Institute, Lahore, and augmented the total compliance rate from 35/60 (58.3%) to 47/60 (78.3%). The SCC was useful in standardizing important safety practices, which is demonstrated by reporting substantial improvements in antibiotic prophylaxis, the use of magnesium sulfate, birth companion support, and early breastfeeding. Low levels of adherence consistently observed in maternal HIV management and discharge planning can be the targets of concentrated efforts, including stigma-reduction training, electronic reminders, and checklist champions. 

Audit-feedback cycles, leadership support, and the incorporation of the SCC into regular work processes must be undertaken indefinitely to maintain these gains and minimize unnecessary maternal and neonatal damage in resource-limited environments.
